# *Diplomonorchis fallax* n. sp. (Digenea: Monorchiidae) from the northern Gulf of Mexico with evaluation of sympatric congeners

**DOI:** 10.1007/s11230-024-10196-7

**Published:** 2024-11-14

**Authors:** Stephen S. Curran, Peter D. Olson, Stephen A. Bullard

**Affiliations:** 1 Aquatic Parasitology Laboratory and Southeastern Cooperative Fish Parasite and Disease Laboratory, School of Fisheries, Aquaculture, and Aquatic Sciences, College of Agriculture, Auburn University, 559 Devall Drive, Auburn, AL 36832 USA; 2 Department of Life Sciences, Natural History Museum, Cromwell Road, London, SW7 5BD UK

## Abstract

*Diplomonorchis micropogoni* Nahhas & Cable, 1964 was considered a junior subjective synonym of *Diplomonorchis leiostomi* Hopkins, 1941 in 1969. *Diplomonorchis leiostomi* has since been widely reported from the coastal Western Atlantic between Delaware Bay and southern Brazil. Until now, taxonomically verifiable DNA sequence data for *D*. *leiostomi* has been available from an individual worm collected from the northern Gulf of Mexico. We generated a partial sequence of the 28S rRNA gene from *D*. *leiostomi* from the spot croaker, *Leiostomus xanthurus* Lacepède (type-host) from Beaufort, North Carolina, USA (type-locality) that differed at 31 of 1,246 bases from the available 28S sequence. This prompted a reevaluation of *Diplomonorchis* spp. identities from the northern Gulf of Mexico. We found *D*. *leiostomi* and *D*. *micropogoni* distinguishable by testes shape and size, and to a lesser degree by relative caecal length. Museum specimens of *D*. *leiostomi*, identified from the Gulf of Mexico represent a species complex containing *D*. *leiostomi*,* D*. cf. *micropogoni* and, a new species of *Diplomonorchis*. The sequences previously identified as *D*. *leiostomi* in GenBank (AY222137 & AY222252) are herein identified as *D*. cf. *micropogoni*. The new species is described from newly collected material herein. Phylogenetic analysis of 28S rRNA sequences from the species complex plus 46 species from the Monorchioidea Odhner, 1911 indicated all three *Diplomonorchis* spp. are closely related and form a clade with some species of *Lasiotocus* Looss, 1907. With the addition of the new species, and acceptance of *D*. *micropogoni*, there are currently 14 valid species in *Diplomonorchis*.

## Introduction

*Diplomonorchis* Hopkins, 1941 was erected for monorchiid digeneans having two well-separated opposing testes situated entirely in the hindbody, and irregularly shaped vitelline follicles distributed in paired groups in the gonadal region of the hindbody. Hopkins (1941) designated *Diplomonorchis leiostomi* Hopkins, 1941 as type-species, which he described from the spot croaker, *Leiostomus xanthurus* Lacepède (type-host) and the pigfish, *Orthopristis chrysoptera* Linnaeus, from Atlantic coastal waters at Beaufort, North Carolina, USA. Subsequently, *D*. *leiostomi* has been reported to infect a variety of coastal fishes in the western Atlantic Ocean (see Table 1) ranging from Delaware Bay (see Thoney, 1993) to coastal Brazil (see Kohn et al., 2007). Hopkins (1941) also created a new combination for a second species, *Diplomonorchis bivitellosus* (Manter, 1940) Hopkins, 1941, which Manter (1940) had originally described from the halfspotted tonguefish, *Symphurus atramentatus* Jordan & Bollman from the Pacific Ocean near the Galápagos Islands. These were the only two accepted species in the genus until the middle of the 1960s when a proliferation of marine parasite studies in the warm Western Atlantic Ocean led to the discovery of more diversity in the genus. *Diplomonorchis hopkinsi* Nahhas & Cable, 1964, *Diplomonorchis micropogoni* Nahhas & Cable, 1964, *Diplomonorchis sphaerovarium* Nahhas & Cable, 1964, and *Diplomonorchis myrophitis* Nahhas & Cable, 1964 were described from coastal Jamaica, and *Diplomonorchis floridensis* Nahhas & Powell, 1965 was described from the Florida, USA Gulf Coast (Nahhas & Cable, 1964; Nahhas & Powell, 1965; Table 1). With the further additions of *Diplomonorchis alexanderi* (Arai, 1963) Kumar, 1997 from the Gulf of California, Mexico; *Diplomonorchis caballeroi* (Zhukov, 1983) Gibson, 2013 from the Gulf of Mexico; *Diplomonorchis catarinensis* Amato, 1982 from coastal Brazil; *Diplomonorchis cumingiae* (Martin, 1938) Amato, 1982 from coastal Massachusetts, USA; *Diplomonorchis kureh* Machida, 2005 from the Western Pacific Ocean; and *Diplomonorchis magnacetabulum* (Thomas, 1959) Overstreet, 1969 from coastal Ghana, Africa, global diversity of *Diplomonorchis* has swollen to 13 species with available names (Table 1). *Diplomonorchis alykhani* Ibrahim, et al., 2022, a digenean recently described from the stomach of the square-tailed mullet, *Ellochelon vaigiensis* (Quoy & Gaimard), from Pakistan, is unrecognizable as a monorchiid based on the description and is not represented by type specimens in a lending museum (Ibrahim et al., 2022). Consequently, *D*. *alykhani* is a *nomen nudum*.

**Table 1:** *Diplomonorchis* spp. with their reported definitive hosts, distributions, and associated records.

Species	Type host	Other host(s)	Geographic range	Record(s)
*D*. *alexanderi* (Arai, 1963) Kumar, 1997	*Paralabrax clathratus* (Girard)		Eastern Pacific Ocean, Mexico	Arai, 1963
*D*. *bivitellosus* (Manter, 1940) Hopkins, 1941	*Symphurus atramentatus* Jordan & Bollman	*Symphurus plagiusa* (L.)	Eastern Pacific Ocean, Galapagos Islands Beaufort, North Carolina	Manter, 1940 Pearse, 1949
*D*. *caballeroi* (Zhukov, 1983) Gibson, 2013	Unidentified *Syacium* sp.		Gulf of Mexico	Zhukov, 1983
	*Syacium papillosum* (L.)		Gulf of Mexico	Vidal-Martínez et al., 2019
*D*. *catarinensis* Amato, 1982	*Micropogonias furnieri* (Desmartest)	*Chaetodipterus faber* (Brousssonet)	Western Atlantic Ocean, Brazil	Amato, 1982
*D*. *cumingae* (Martin, 1938) Amato, 1982	Unidentified “flounders and eels”		Woods Hole, Massachusetts	Martin, 1940
*D*. *floridensis* Nahhas & Powell, 1965	*S*.* plagiusa*		Gulf of Mexico, Florida	Nahhas & Powell, 1965
		Unidentified *Symphurus* sp.	Western Atlantic Ocean, Brazil	Wallet & Kohn, 1987
*D*. *hopkinsi* Nahhas & Cable, 1964	*M*.* furnieri*		Caribbean Sea, Jamaica	Nahhas & Cable, 1964
*D*. *kureh* Machida, 2005	*Diagramma pictum* (Thunberg)		Western Pacific Ocean, Japan	Machida, 2005
		*Diagramma labiosum* MacLeay	Western Pacific Ocean, Queensland, Australia	Searle et al., 2014
*D*. *leiostomi* Hopkins, 1941	*Leiostomus xanthurus* Lecepède	*Orthopristis chrysoptera* (L.)	Western Atlantic Ocean, Beaufort, North Carolina	Hopkins, 1941
		*Pogonias cromis* (L.)	Gulf of Mexico, Louisiana	Sparks, 1958*
		*Bairdiella chrysura* (Lecepède), *Lagodon rhomboides* (L.)	Gulf of Mexico, Florida	Sogandares-Bernal & Hutton, 1959*
		*O*. *chrysoptera*, *L*. *rhomboides Micropogonias undulatus* (L.), *Monacathus hispidus* (L.), *L*.* xanthurus*	Gulf of Mexico, Florida	Nahhas & Powell, 1965*
		*Trinectes maculatus* (Bloch & Schneider)	Gulf of Mexico, Louisiana	Corkum, 1966*
		*Archosargus rhomboidalis* (L.)*, L*. *rhomboides*, *O*. *chrysoptera*	Biscayne Bay, Florida	Overstreet, 1969*
		*Haemulon Sciurus* (Shaw)	Western Atlantic Ocean, Brazil	Kohn et al., 1982
		*Boridia grossidens* Cuvier	Western Atlantic Ocean, Brazil	Fernandes et al., 1985
		*Haemulon steindachneri* (Jordan & Gilbert), *Orthopristis rubra* Cuvier	Western Atlantic Ocean, Brazil	Luque et al., 1996
		*M*. *furnieri*	Western Atlantic Ocean, Brazil	Alves & Luque, 1999; Alves & Luque, 2001
*D*. *magnacetabulum* (Thomas, 1959) Overstreet, 1969	*Cynoglossus senegalensis* (Kaup)		Eastern Atlantic Ocean, Ghana	Thomas, 1959
*D*. *micropogoni* Nahhas & Cable, 1964	*M*.* furnieri*	*A*. *rhomboidalis* *L*. *xanthurus* *L*. *rhomboides*, *L*. *xanthurus*, *M*. *undulatus*	Caribbean Sea, Jamaica Northern Gulf of Mexico Northern Gulf of Mexico	Nahhas & Cable, 1964 Olson et al., (2003) Present study
		*Stellifer lanceolatus* (Holbrook)	Western Atlantic Ocean, Sapelo Island, Georgia	Present study
*D*. *myrophitis* Nahhas & Cable, 1964	*Myrophis punctatus* Lütken		Caribbean Sea, Jamaica	Nahhas & Cable, 1964
*D*. *sphaerovarium* Nahhas & Cable, 1964	*Sphoeroides testudineus* (L.)		Caribbean Sea, Jamaica	Nahhas & Cable, 1964
		*Ophichthus gomesii* (Castelnau)	Biscayne Bay, Florida	Overstreet, 1969
		*O*. *gomesii*	Western Atlantic Ocean, Brazil	Fernandes et al., 2002

*We consider these reports to represent in full or in part *D*. cf. *micropogoni*.

Prior to this study, available taxonomically verifiable molecular data for *Diplomonorchis* was limited to two sequences in GenBank: the complete small subunit ribosomal RNA (18S rRNA) gene (AY222137), and partial large subunit rRNA gene including variable domains D1–D3 (partial 28S rRNA gene) (AY222252). These were sourced from an individual worm identified as *D*. *leiostomi* collected from *L*. *xanthurus* from coastal Mississippi, USA, and used in a large phylogenetic study of the Digenea (Olson et al., 2003). Identification of the material used in the Olson et al. (2003) study as *D*. *leiostomi* followed the conventionally accepted opinion by Overstreet (1969) that there was overlap in key morphological features in *D*. *micropogoni* and *D*. *leiostomi*. *Diplomonorchis micropogoni* was considered a junior synonym of *D*. *leiostomi* by Overstreet (1969), but we herein propose the acceptance of *D*. *micropogoni* and explain this opinion in the results below.

In the summer of 2018, we opportunistically collected two specimens of *D*. *leiostomi* from *L*. *xanthurus* in Beaufort, North Carolina, USA and generated a fragment of the partial 28S rRNA gene and aligned it with AY222252 for comparison. The North Carolina sequence differed substantially from the Mississippi sequence, indicating they do not represent the same species. This inconvenient fact prompted a reassessment of the identity of the material used in the Olson et al. (2003) study and some other specimens belonging in *Diplomonorchis* collected from the Gulf of Mexico. Morphological observations of museum specimens and newly collected specimens combined with newly generated ribosomal nucleotide sequence data revealed a species complex in the Gulf of Mexico. A previously unidentified *Diplomonorchis* sp. is described as a new species, and a second species previously confused with *D*. *leiostomi* (e.g., Olson et al., 2003; Panyi et al., 2020) is recognized and described as *D*. cf. *micropogoni*. We consider the newly generated sequence from North Carolina to represent *D*. *leiostomi sensu stricto* and supplemental data from the second specimen of *D*. *leiostomi* from North Carolina is provided. No new molecular data from material from the Gulf of Mexico was genetically identical with *D*. *leiostomi sensu stricto*, however, some of the museum vouchers labeled *Diplomonorchis* sp. from Galveston Bay, Texas are morphologically consistent with *D*. *leiostomi sensu stricto* and morphological data are provided from these. The phylogenetic relationships of the three studied *Diplomonorchis* spp. are evaluated using Bayesian Inference analysis of new and publicly available 28S rRNA gene fragments.

## Materials and methods

### Collections

Digeneans used in the study were collected from various hosts and localities as indicated. Numerous specimens of *Diplomonorchis* spp. were collected from *L*. *xanthurus* from Biloxi Bay, Mississippi, USA (30° 23ʹ 19.74″ N, 88° 49ʹ 33.22″ W) using otter trawl (14–18 February 2005; 25 April 2005), from Mississippi Sound (30° 18ʹ 13.05″ N, 88° 41ʹ 49.26″ W) using otter trawl (3 November 2020), and from Mobile Bay (30° 25ʹ 56.59″ N, 88° 03ʹ 27.07″ W) using gillnet (23 February 2021, 8 March 2021). Numerous specimens of *Diplomonorchis* spp. were collected from the Atlantic croaker, *Micropogonias undulatus* (Linnaeus) from the northern Gulf of Mexico (30° 13ʹ 00.96″ N, 88° 42ʹ 45.26″ W) using otter trawl (21 October 2010), and Mobile Bay (30° 34ʹ 58.91″ N, 87° 56ʹ 38.22″ W) using gillnet (23 March 2021) and otter trawl (28 April 2022). Numerous specimens of *Diplomonorchis* sp. were collected from specimens of the pinfish, *Lagodon rhomboides* (Linnaeus) from Gulf Breeze, Florida, USA (30° 21ʹ 15.28″ N, 87° 09ʹ 30.03″ W) by seine (23 June 2016). Two specimens of *Diplomonorchis* sp. were collected from the American stardrum, *Stellifer lanceolatus* (Holbrook) from near Sapelo Island, Georgia, USA (31°23ʹ11.05″ N, 81°17ʹ32.63″ W) by otter trawl (31 March 2007). Two specimens of *D*. *leiostomi* were collected from *L*. *xanthurus* from Beaufort, North Carolina, USA (34° 41ʹ 52.66″ N, 76° 40ʹ 32.75″ W) by hook and line (3 June 2018).

### Specimen preparation and morphological study

Fish were either dead upon collection from trawls or euthanized by cranial pithing after sedation in seawater with added tricaine methanesulfonate. Digestive tracts were removed from each studied fish and immersed in saline solution (0.85% sodium chloride in distilled water). Digestive tracts were cut open, worms were removed and rinsed in saline solution, and observed using a dissecting microscope. Worms were divided into lots destined for DNA extraction or morphological study. Those for morphological study were killed by immersion in near boiling tap water and fixed in 10% formalin solution. Formalin-fixed worms were later stained using aqueous hematoxylin solution (Van Cleave’s plus Ehrlich’s hematoxylin) and mounted on glass slides following the procedure outlined in Curran et al. (2022). Monorchiids studied were assigned to *Diplomonorchis* because they had two separated symmetrical testes situated in the middle third of the hindbody, vitelline follicles distributed in the gonadal region, and an I-shaped excretory bladder (Madhavi, 2008). Drawings were made by digitizing sketches made using a camera lucida. Measurements were obtained using a Jenoptik Gryphax camera and software (Jena, Germany) with an Olympus BX51 microscope equipped with differential interference contrast optical components (Center Valley, Pennsylvania, USA). Specimens of described species are deposited in the Smithsonian Institution National Museum of Natural History (USNM) Invertebrate Zoology Collection, Washington, DC, and in the British Museum of Natural History, London, UK (NHML), with accession numbers provided in the results section. Museum specimens were borrowed for comparison from USNM, NHML, and the Harold W. Manter Laboratory of Parasitology (HWML), Lincoln, Nebraska, USA. Borrowed specimens were: two specimens of *D*. *bivitellosus* (USNM 1322095, 1337793), one specimen of *D*. *hopkinsi* (USNM 1356063), three specimens identified as *D*. *leiostomi* (USNM 1355872, 1380451, 1380447), four specimens identified as *Diplomonorchis* sp. (USNM 1398097, 1398098), two specimens identified as *D*. *leiostomi* (NHML 2003.2.11.1-2), two paratypes of *D*. *catarinensis* (HWML 21325), five specimens identified as *D*. *leiostomi* (HWML 863, 22286, 38300), and 38 specimens identified as *Diplomonorchis* sp. (HWML 38301, 38302). Measurements from collected material and some of the museum vouchers are provided in Table 2.

**Table 2:** Morphometric data of *Diplomonorchis* spp. All direct measurements in micrometers. Metrix expressed in ranges when appropriate, as ratios or percentages when appropriate. Means are provided in parentheses when appropriate.

Species	*D*. *leiostomi*	*D*. *Leiostomi*	*D*. cf. *leiostomi*	*D. micropogoni*	*D*. cf. *micropogoni*	*D.* cf.* micropogoni*	*D*. cf. *micropogoni*	*D*. cf. *micropogoni*	*D.* cf.* micropogoni*	*D.* cf.* micropogoni*	*D.* cf.* micropogoni*	*D.* cf.* micropogoni*	*D*. *fallax* n. sp.	*D*. *fallax* n. sp.
Original identificaiton	*D*. *leiostomi*		*Diplomonorchis* sp.	*D. micropogoni*					*D*. *leiostomi*	*D*. *leiostomi, Diplomonorchis* sp.	*D*. *leiostomi*	*D*. *leiostomi*		*D*. *leiostomi*
Number of specimens	136	1	39	17	24	9	4	2	1	5	3	2	32	1
Host(s)	*Leiostomus xanthurus*, *Orthopristis chrysopterus*	*L*. *xanthurus*	*Micropogonias undulatus*	*Micropogonias furnieri*, *Archosargus rhomboidalis*	*M*.* undulatus*	*Lagodon rhomboides*	*L*. *xanthurus*	*Stellifer lanceolatus*	*L*. *xanthurus*	*M*.* undulatus*	*L*. *rhomboides*	*A*. *rhomboidalis*	L.* xanthurus*	L.* xanthurias*
Material source	Original description	New collection	HWML 38301, 38302	Original description	New collections	New collections	New collections	New collection	NHML 2003.2.11.2b,	USNM 1380451, 1398097, 1398098	HWML 22286, USNM 1355872	HWML 863	New collections	NHML 2003.2.11.2a
Locality	Beaufort, North Carolina, USA	Beaufort, North Carolina, USA	Galveston Bay, Texas, USA	Jamaica, Caribbean Sea	Mississippi and Alabama, USA	Florida, USA, Gulf of Mexico	Alabama, USA	Georgia, USA	Mississippi, USA	Matagorda Bay, Texas and Mississippi, USA	Tampa and Apalachee Bays, Florida, USA	Biscayne Bay, Florida, USA	Mississippi and Alabama, USA	Mississippi, USA
Body length (BL)	400–800, 520–477*	313	196–356 (289)	233–634†	417–679 (502)	402–670 (521)	453–566 (519)	356–398	573	333–475 (427)	528–867 (657)	463–748	526–879 (687)	646
Body width (BW)	~(350) 289–306*	225	130–253 (184)	186–435†	241–393 (298)	213–417 (320)	273–311 (292)	175–197	378	171–300 (257)	380–517 (432)	303–335	193–305 (236)	240
BL/BW	1.6–1.8:1* (1.7:1)	1.4:1	1.3–1.7:1 (1.6:1)	~ 1.5:1†	1.5–1.9:1 (1.7:1)	1.5–1.9:1 (1.6:1)	1.6–1.9:1 (1.8:1)	2:1	1.5:1	1.5–1.7:1 (1.6:1)	1.3–1.7:1 (1.5:1)	1.4–1.5:1	2.3–3.5:1 (2.9:1)	2.7:1
Forebody length (FL)	~ 174–206*	106	64–111 (88)	~ 219†	122–202 (153)	142–219 (169)	116–189 (153)	112–128	162	126–168 (144)	158–291 (206)	132–149	176–312 (236)	198
FL/BL	~ 36–40*	34	26–38 (31)	~ 35†	26–34 (31)	27–35 (33)	26–33 (29)	31–32	28	29–38 (34)	27–34 (31)	20–29	25–38 (34)	31
Oral sucker (OS) Length (L)	60–90 (80)	49	33–50 (41)	46–83 (74†)	62–90 (75)	66–86 (79)	53–71 (63)	59–69	63	59–86 (68)	77–88 (84)	68	46–59 (52)	50
OS Width (W)	60–90 (80)	61	36–65 (49)	66–98 (98†)	66–95 (81)	76–91 (84)	60–79 (72)	63–69	81	68–92 (75)	90–108 (99)	84–99	47–60 (53)	54
OS L/BL	~ 12–13*	16	10–19 (14)	~ 12†	12–17 (15)	13–17 (15)	11–13 (12)	17	11	14–20 (16)	10–15 (13)	9–15	6–10 (8)	8
OS W/BW	~ 23–26*	27.1	18–33 (25)	~ 23†	23–30 (28)	20–36 (27)	21–29 (25)	35–36	21	25–31 (28)	21–25 (23)	28–30	18–27 (23)	23
Prepharynx L	0	6	0–5	0	0–20 (7)	0	0–10 (5)	0	0	0	0	0	14–52 (30)	15
Pharynx L	~ 31–40*	32	16–24 (19)	22–37 (28†)	30–43 (37)	40–54 (45)	30–40 (35)	32–34	30	31–38 (34)	44–45 (44)	35–37	28–39 (34)	33
Pharynx W	~ 31–40*	38	17–27 (20)	27–53 (46†)	35–59 (43)	35–48 (45)	32–46 (42)	31–33	36	32–44 (37)	43–47 (45)	38	28–36 (31)	30
Pharynx L/BL	~ 5–10*	10.2	5–9 (7)	~ 4	5–9 (7)	7–11 (9)	6–7 (7)	9	5	7–9 (8)	5–8 (7)	5–8	4–6 (4)	5
Oesophagus L	0–40, 12–18*	0	0–5	0	0–25 (5)	0–10 (3)	0–10 (4)	0	4	0	0–19 (10)	0–15	27–73 (48)	27
Post-caecal L	~ 29–71*	30	11–45 (29)	~ 124*†	37–96 (63)	80–150 (122)	58–78 (70)	45–76	90	40–96 (71)	89–158 (113)	69–74	95–199 (135)	95
Post-caecal L/BL	~ 6–14*	10	5–12 (10)	~ 20†	8–16 (12)	20–31 (24)	13–15 (14)	13–19	16	9–21 (16)	16–18 (17)	9–16	15–23 (19)	15
Ventral sucker (VS) L	40–60 (50)	42	27–50 (36)	37–67 (57†)	51–89 (64)	51–63 (57)	46–68 (57)	45–47	67	40–66 (50)	58–74 (65)	49–56	31–45 (39)	36
VS L/BL	~ 6–9*	13	9–16 (12)	~ 9†	9–15 (13)	8–13 (11)	10–14 (11)	12–13	12	9–15 (12)	9–11 (10)	7–11	4–7 (6)	6
VS W	40–60 (45)	44	28–48 (39)	37–67 (60†)	52–81 (66)	52–65 (57)	47–66 (60)	42–46	61	49–77 (57)	68–74 (71)	55–67	34–44 (38)	35
VS W/BW	~ 14–18*	20	16–28 (21)	~ 14†	17–26 (22)	13–25 (18)	17–24 (20)	23–24	16	14–29 (26)	14–18 (17)	12–18	13–19 (16)	15
OS W: VS W ratio	1:0.6–0.7*	1:0.7	1:0.7–0.9	1:0.6–0.8 (1:0.6†)	1:0.6–0.9	1:0.6–0.7	1:0.8–0.9	1:0.7	1:0.8	1:0.7–0.8	1:0.7–0.8	1:0.6–0.7	1:0.6–0.8	1:0.6
Testes L	60–77*	55–62	32–72	54–166 (156–159†)	79–148	74–136	95–122	91–114	151–173	100–130	105–135	106–135	70–186	101–120
Testes W	60–68*	49–57	25–65	38–80 (64–71†)	59–103	49–96	67–100	51–61	59–84	67–97	69–89	59–67	42–92	53–56
Ratio of teste L: W	1:0.8–1.1*	1:0.9	1:0.7–1.2	1:0.4–0.5†	1:0.6–0.9	1:0.6–0.8	1:0.7–0.8	1:0.4–0.6	1:0.4–0.5	1:0.6–0.7	1:0.7–0.8	1:0.4–0.6	1:0.4–0.8	1:0.5
Posttesticular space L	~ 109–126*	63	30–113 (77)	~ 135†	90–177 (126)	80–217 (123)	94–146 (117)	67–91	125	78–116 (97)	83–172 (121)	75–106	103–205 (144)	147
Posttesticular space/BL	~ 23–24*	20	15–40 (27)	~ 21†	20–30 (25)	20–32 (25)	19–26 (22)	19–23	22	18–24 (21)	16–20 (18)	10–23	16–27 (21)	23
Egg L	22–30	27–31	23–30	22–30	20–29 (25), n=57	24–30 (29), n=29	20–26 (24), n=15	21–22	27–30	19–28	24–31	27–30	17–23 (20), n=60	22–23
Egg W	14–20	15–16	13–21	14–18	13–19 (15), n=54	13–18 (16), n=26	13–19 (13), n=13	11–12	18–19	14–18	16–20	18–19	11–14 (13), n=52	13–16

Abbreviations: L, length; W, width; *=estimated from description; †=estimated from holotype drawing in description.

### DNA extraction, amplification and sequence generation

Live worms slated for molecular analysis were submerged individually in vials with 95% ethanol. Genomic DNA was extracted from these worms using a DNeasy Blood and Tissue kit (Qiagen Incorporated, Valencia, California, USA) following the included protocol. The partial 28S rRNA gene was generated for all *Diplomonorchis* spp. studied. Additionally, the internal transcribed spacer regions 1 (ITS1) and 2 (ITS2) were generated for 2 *Diplomonorchis* spp. studied (frozen genomic DNA from *D*. *leiostomi sensu stricto* was lost before we were able to amplify ITS1 and ITS2 from the single replicate).

Polymerase chain reactions (PCR) were used to amplify the three DNA fragments using distinct primer sets. A DNA fragment spanning the complete ITS1 region was amplified using forward primer 18S-ITS1 (5′-CCGTCGCTACTACCGATTGAA-3′) and reverse primer 5.8S-ITS1 (5′-CGCAATGTGCGTTCAAGATGTC-3′) (Bagnato et al., 2016). A DNA fragment spanning the ITS2 region was amplified using forward primer 3S (5′-GGTACCGGTGGATCACGTGGCTAGTG-3′) (Morgan & Blair, 1995) and reverse primer ITS2.2 (5ʹ-CCTGGTTAGTTTCTTTTCCTCCGC-3ʹ) (Cribb et al., 1998). A portion of the 28S rRNA gene at the 5ʹ end was amplified using forward primer LSU5 (5ʹ-TAGGTCGACCCGCTGAAYTTAAGCA-3′ (Littlewood, 1994) and reverse primer 1500R (5′-GCTATCCTGAGGGAAACTTCG-3′) (Snyder & Tkach, 2001). PCR was performed using 50 µl sample volumes containing 10 µl 5X Taq buffer (Promega Corporation, Madison, Wisconsin, USA), 1 µl 10 µM dNTP (Promega Corporation), 1 µl 10 µM forward primer, 1 µl 10 µM reverse primer, 0.3 µl GoTaq DNA polymerase (Promega Corporation), 2 µl of extracted DNA, with ~ 35 µl purified water. PCR products were then purified using the QIAquick PCR purification kit (Qiagen Incorporated). Purified DNA sample concentration was estimated using a Nano-Drop-1000 spectrophotometer (Thermo Scientific Corporation, Nanodrop Technologies, Waltham, Massachusetts, USA). Purified DNA samples were prepared for 15 µl Sanger sequencing reactions (2.5 µl 10 µM primer + purified DNA + purified water), with DNA volume and water volume depending on DNA sample concentration. Additional forward primer 300F (5ʹ-CAAGTACCGTGAGGGAAAGTTG-3ʹ) and reverse primer ECD2 (5′-CTTGGTCCGTGTTTCAAGACGGG-3′) (Littlewood et al., 1997) were used for increasing sequence coverage for the partial 28S rRNA fragment during Sanger sequencing which was outsourced to GENEWIZ (Azenta Life Sciences, South Plainfield, New Jersey, USA). Generated sequence fragments were assembled using MAFFT (Katoh & Standley, 2013) in Geneious Prime Software version 2022.0.2 (Geneious Corporation, Auckland, New Zealand). Assembled sequences were aligned and compared using BioEdit version 7.2.5 software (Hall, 1999). The aligned ITS1 and ITS2 regions were used strictly for comparative purposes and were not analyzed phylogenetically. Representative nucleotide sequences from *Diplomonorchis* spp. are deposited in GenBank under accession numbers provided in the results section.

### Molecular phylogeny

We aligned the partial 28S rRNA gene sequences from the three studied *Diplomonorchis* spp. with 46 other sequences from GenBank, all from digeneans belonging in the Monorchioidea Odhner, 1911 and used this to conduct Bayesian Inference analysis. The ingroup consisted of 5 species from the Lissorchiidae Magath, 1917 and 43 species from the Monorchiidae Odhner, 1911. *Skrjabinopsolus nudidorsalis* Sokolov et al., 2020 served as the outgroup for the analysis and was chosen based on its classification in the Deropristidae Cable & Hunninen, 1942, basal within the Monorchioidea as previously determined and demonstrated by Sokolov et al. (2020). The alignment was trimmed to 1,257 nucleotide bases to match the shortest sequence. JModelTest 2 version 2.1.10 was used to determine the best nucleotide substitution model, which was determined to be the general time reversible model with gamma-distribution rate variation across sites and a proportion of invariable sites (Darriba et al., 2012). Phylogenetic analyses were conducted using MrBayes software version 3.2.7 (Ronquist and Huelsenbeck, 2003) with gaps in the alignment treated as missing data. Markov chain Monte Carlo (MCMC) chains were run for 3,000,000 generations with sample frequency set at 1,000. A consensus tree was obtained with burn-in fraction set at 0.25 when the average standard deviation of split frequencies descended below 0.002. The consensus tree was visualized using FigTree software version 1.4.3 (Rambaut et al., 2014).

## Results

### Nucleotide sequence data

The partial 28S rRNA fragment from *D*. *leiostomi* from North Carolina, USA was 1,313 bases in length. When aligned with the 1,246 base long sequence AY222252, a sequence previously submitted to GenBank and identified as *D*. *leiostomi* from Mississippi, USA (see Olson et al., 2003), the North Carolina sequence differed from the Mississippi sequence by 9 base insertions and 22 single nucleotide changes. The North Carolina voucher was morphologically consistent with the original description of *D*. *leiostomi* and prompted us to identify its corresponding sequence as *D*. *leiostomi sensu stricto*. The genomic DNA from *D*. *leiostomi sensu stricto* was stored in freezers that were lost during the study period and we were unable to amplify other gene regions.

Additional sequences used in the study were sourced from individual worms, with numbers of sequence replicates for each digenean species studied provided below. The 28S rRNA, ITS1 and ITS2 were generated from two other *Diplomonorchis* spp. during the study. No intraspecific variation was observed for these two species. Morphological observations of the new material revealed that one of the species was consistent with the description for *D*. *micropogoni* and the associated 28S rRNA fragment was 100% identical with AY222252. The other *Diplomonorchis* sp. represented an undescribed species. Examination of the two museum vouchers associated with AY222252 (NHML 2003.2.11.1-2) further confounded the problem: one voucher was consistent with *D*. *micropogoni* (Now accessioned NHML 2003.2.11.2b) whereas the other was consistent with the undescribed *Diplomonorchis* sp. (Now accessioned NHML 2003.2.11.2a). Based on our newly collected material and generated sequences, we herein identify AY222252 and one of the NHML vouchers as *Diplomonorchis* cf. *micropogoni*. Our new genetic isolates of *D*. cf. *micropogoni* were from *L*. *xanthurus* (3 worms) and *M*. *undulatus* (8 worms). Genetic isolates of the undescribed species were from *L*. *xanthurus* (8 worms). The undescribed species is described below.

Comparison of a 1,246 bp alignment of the partial 28S rRNA sequences from *D*. *leiostomi sensu stricto* from North Carolina, USA, *D*. cf. *micropogoni*, and the new species, revealed divergence ranging from 1.4% between* D*. cf. *micropogoni* and the new species, to 2.5% between* D*. cf. *micropogoni* and *D*. *leiostomi sensu stricto* (Table 3). Comparison of a 733 bp alignment of the ITS1 regions between *D*. cf. *micropogoni* and the new species revealed 1.2% variation (Table 4). *Diplomonorchis* cf. *micropogoni* and the new species were identical over 419 aligned bases spanning the complete ITS2 region.

**Table 3:** Pairwise comparison of partial ribosomal rRNA regions of 3 *Diplomonorchis* spp. Numbers indicate deletions, followed by point mutations and % variation in parentheses. The 28S rRNA fragment (1,246 aligned bases) is compared above the diagonal.

Species	*D*.* leiostomi*	*D*. *fallax* n. sp.	*D*. cf. *micropogoni*
*D*.* leiostomi*	–	0, 22, (1.8%)	9, 22, (2.5%)
*D*. *fallax* **n. sp.**		–	9, 8, (1.4%)
*D*. cf. *micropogoni*			–

**Table 4:** Pairwise comparison of internal transcribed spacer regions 1 and 2 between two *Diplomonorchis* spp. Numbers indicate deletions, followed by point mutations and % variation in parentheses. The ITS1 region (733 aligned bases) is compared above the diagonal and the ITS2 (419 aligned bases) is compared below the diagonal. ITS data is not available for *Diplomonorchis leiostomi* Hopkins, 1941.

Species	*D*. *fallax* n. sp.	*D*. cf. *micropogoni*
*D*. *fallax* **n. sp.**	–	0, 9, (1.2%)
*D*. cf. *micropogoni*	0, 0, (0%)	–

## Descriptions and supplemental morphological data

**Fig. 1:**
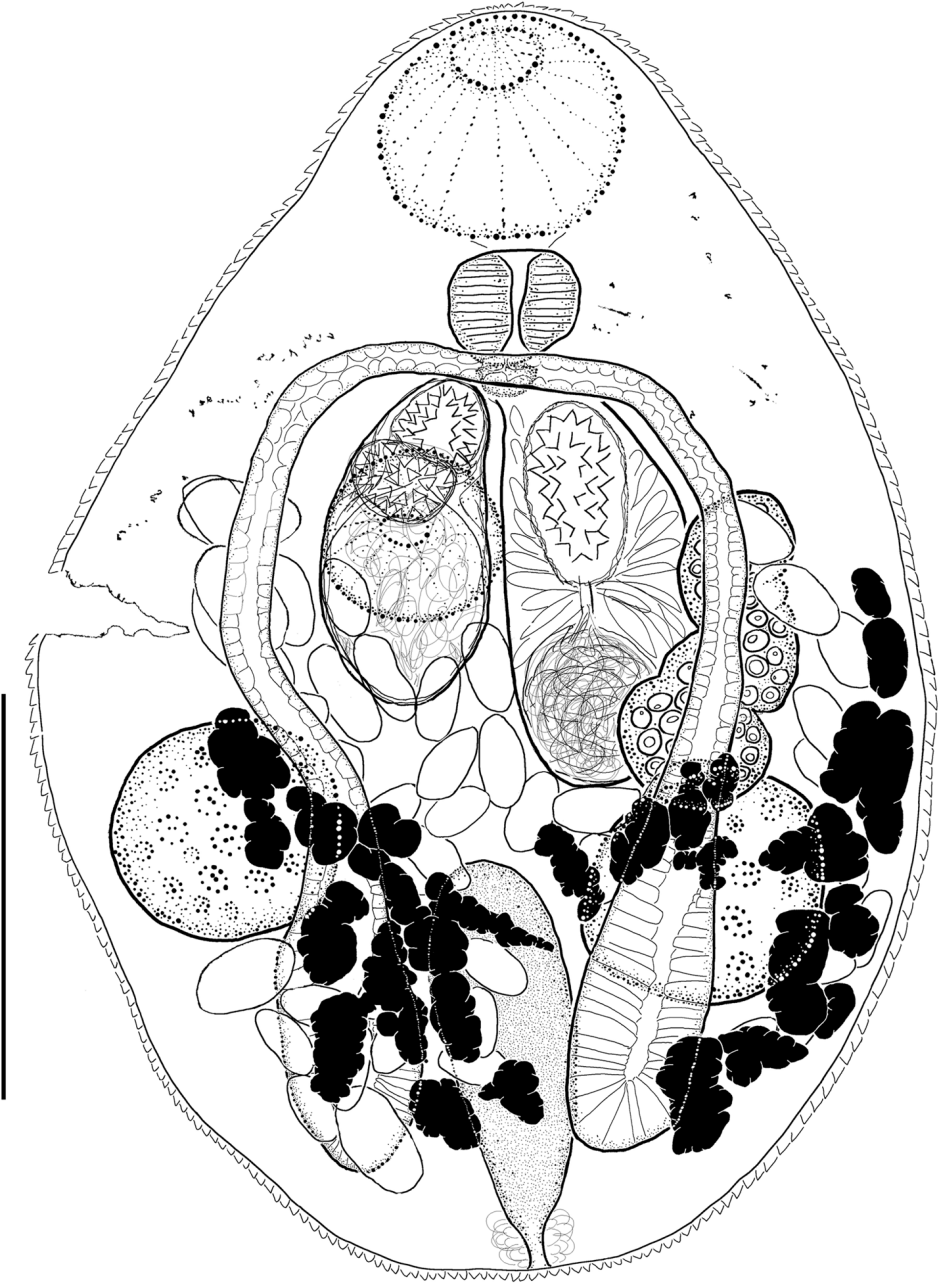
*Diplomonorchis leiostomi* Hopkins 1941. Dorsal view of adult collected from *Leiostomus xanthurus* Lacepède in Beaufort, North Carolina, USA. Scale bar: 100 µm.

***Diplomonorchis leiostomi*** **Hopkins, 1941**

(Figs. 1, 4A; Table 2).

*Type-host: Leiostomus xanthurus* Lacepède, spot croaker.

*Type-locality:* Beaufort, North Carolina, USA.

*Other hosts and localities from reports: Orthopristis chrysoptera* (Linnaeus), pigfish, Beaufort, North Carolina, USA (Hopkins, 1941); *L*. *xanthurus* and *Micropogonias undulatus* (Linnaeus), Atlantic croaker, Atlantic Ocean, USA (Thoney, 1991; 1993); *Haemulon Sciurus* (Shaw), bluestriped grunt, *Boridia grossidens* Cuvier, borriqueta porgy, *Haemulon steindachneri* (Jordan & Gilbert), chere-chere grunt, *Orthopristis rubra* (Cuvier), corocoro grunt, and *Micropogonias furnieri* (Desmarest), whitemouth croaker, Atlantic Ocean, Brazil (Kohn et al., 1982; Fernandes et al., 1985; Alves & Luque, 1999; 2001).

*Other hosts and localities confirmed by museum vouchers: M. undulatus* from Galveston Bay, Texas, USA (HWML 38301, 38302).

*Host and locality of 2 specimens studied: L*. *xanthurus*, Beaufort, North Carolina, USA, (34° 41ʹ 52.66″ N, 76° 40ʹ 32.75″ W).

*Site of infection:* Intestine.

*Prevalence and intensity:* 2 worms infected one of two fish examined (3 June 2018).

*Specimen deposited:* 1 Voucher (USNM 1739551).

*Sequence data deposited in GenBank:* partial 28S rRNA, 1 isolate (PQ336760).

### Supplemental data

(Based on 1 specimen). Body ovoid. Tegument spined. Mouth subterminal. Oral sucker subspherical. Ventral sucker subspherical, smaller than oral sucker, in anterior body half. Prepharynx very short. Pharynx subspherical. Oesophagus absent. Intestine bifurcating midway between suckers. Caeca extending well into post-testicular zone, blind. Forebody containing diffuse remnants of eyespots.

Testes two, nearly spherical, slightly oblique, partially straddling caeca and extending far into extracaecal space in middle third of ventral hindbody. Cirrus sac claviform, 108 µm long by 32 µm wide, extending posteriorly into ovarian zone and reaching to anterior margin of right testis, containing proximal ovoid internal seminal vesicle (36 µm long by 32 µm wide), narrow, straight prostate duct surrounded by dense prostate cells, and distal cirrus. Cirrus occupying about 1/3 the length of the entire cirrus sac; spines triangular (3–6 µm long per side).

Ovary 117 µm long by 50 µm wide, lobed, dextral in anterior hindbody; posterior margin overlapping with anterior margin of right testis. Mehlis’ gland, Laurer’s canal, and seminal receptacle not observed. Uterus coiling throughout most of hindbody, with coils filling inter-testicular and post-testicular hindbody. No coils ascending into forebody. Junction of metraterm and terminal organ not observed. Terminal organ bipartite (75 µm long by 54 µm wide), slightly smaller than cirrus sac; proximal portion sac-like, unspined, thin walled; distal portion bending in dorso-ventral plane, thick-walled, lined internally with triangular spines (3–6 µm long per side). Genital atrium median, lacking spines. Genital pore median in ventral forebody, at level of intestinal bifurcation (centered 11 µm anteriad from ventral sucker). Vitellarium comprising 2 groups of irregular follicles. Follicles mostly dorso-median to testes but extending laterally beyond testes, anteriorly into ovarian zone, and into posttesticular zone.

Excretory vesicle I-shaped, extending anteriorly in dorsal hindbody into inter-testicular zone. Pore terminal.

### Remarks

The holotype for *D*. *leiostomi* (USNM 1337478) is the only specimen of the species from the coastal Atlantic Ocean of USA in a lending museum. All other available museum vouchers deposited in either the NHML or the two major lending museums in the USA (i.e. USNM and HWML) identified as *D*. *leiostomi* originate from the Gulf of Mexico. The two specimens of *D*. *leiostomi sensu stricto* we recovered from the type-host and locality in North Carolina are therefore critical for understanding the original concept of the species. We identified our specimen from North Carolina as *D*. *leiostomi* based primarily on the ovoid body shape, sucker width ratio, posterior extent of caeca, testes size, shape and position, and egg size (Table 2). The ratio of the oral to ventral sucker width is 1:0.7 in our specimen, compared with 1: 0.6–0.7 in the original description. Post-caecal space is 10% of body length in our specimen and we calculated this to range from about 6–14% in illustrations in the original description. The testes are nearly spherical, measuring 55–62 µm long by 49–57 µm wide in our specimen, compared with 60–77 µm long by 60–68 µm wide in the original description. Post-testicular space is 20% of body length in our specimen, and Hopkins (1941) described the testes as being about halfway between the ventral sucker and the posterior body end. We estimated the post-testicular space to be 23–24% of body length in illustrations in the original description. The eggs are 27–31 µm long by 15–16 µm wide in our specimen, compared with 22–30 µm long by 14–20 µm wide in the original description. Additionally, the male and female terminal genitalia of our specimen conform to the described configurations by Hopkins (1941), and spine sizes for the cirrus and terminal organ were the same shape and as in the original description (triangular spines measuring ~3–6 µm per side verses ~ 6 µm per side reported by Hopkins [1941]). The ovary is clearly lobed in our specimen, but we could not determine the number of lobes because eggs obscured the view. The ovary was described as divided into three distinct lobes by Hopkins (1941).

Our specimen of *D*. *leiostomi* differed slightly from the original description in overall body size, oral sucker length, and anterior extent of the excretory vesicle (Table 2). Our specimen is smaller in body length (313 µm) and width (225 µm) than the average measurements reported from the original material (600 µm long by 350 µm wide), and the oral sucker length is relatively short (49 µm long compared with 60–90 µm long) (Hopkins, 1941). The excretory vesicle of our specimen extends anteriorly to the mid-testicular level, which is 13 µm (4% of body length) below the posterior margin of the ovary. Hopkins (1941) reported that the excretory bladder extends to the posterior margin of the ovary in the original description. Despite these minor inconsistencies between our specimen and the original description, we value the sucker width ratio, egg size, and especially testes size and shape, as the most important features useful for distinguishing *D*. *leiostomi* from its most similar congeners and based on these key features we are confident that our material represents the same species that Hopkins studied. We suspect the small size of our specimen may be a result of it being recently acquired by the host, and that the minor divergences in oral sucker length and excretory bladder length may be associated with the development of the specimen or excessive pressure during the mounting process.

During our evaluation of the morphology and novel nucleotide data from our North Carolina material and examining museum specimens and new material from the Gulf of Mexico, we discovered that some of the museum specimens identified as *Diplomonorchis* sp. from Texas, USA are consistent with our concept of *D*. *leiostomi* but all of the specimens identified as *D*. *leiostomi* are misidentified and conformed largely or wholly to the description for *D*. *micropogoni* or the new species. The specimens consistent with *D*. *leiostomi sensu stricto* are contained in two specimen lots (HWML 38301, 38302), collectively containing 40 specimens collected from Atlantic croakers (*M*. *undulatus*) from Galveston Bay, Texas, USA in 1970 by Wilbur L. Bullock. We herein identify Bullock’s specimens as *D*. cf. *leiostomi* and retain the confer abbreviation in the name until nucleotide data can be employed to verify the name, and because the specimens are collectively much smaller than specimens from the Atlantic Coast of USA. Bullock’s specimens from Texas are mostly even smaller in body size (196–356 µm long by 130–253 µm wide, mean size 289 µm long by 184 µm wide) than our specimen from North Carolina (313 µm long by 225 µm wide) and the original material of Hopkins (1941) (400–800 µm long by 250–450 µm wide). However, different size classes of *Diplomonorchis* sp., even within an individual host, are not unprecedented and might be attributed to a host periodically feeding on bivalves heavily infected with metacercariae (Martin, 1940; Nahhas & Cable, 1964; Nahhas & Powell, 1965; Overstreet, 1969). The key features that align the Texas specimens with *D*. *leiostomi* from North Carolina are: ratio of oral to ventral sucker width (1:0.7–0.9 compared with 1:0.6–0.7), a short post-caecal space (5–12% of body length compared with 6–14), small nearly spherical testes (32–72 µm long by 25–65 µm wide compared with 60–77 µm long by 60–68 µm wide), ratio of testis length to width (1:0.7–1.2 compared with 1:0.8–1.1), and egg size (23–30 µm long by 13–21 µm wide compared with 22–31 µm long by 14–20 µm wide). Some measurements from these specimens are provided in Table 2. The misidentified specimens we refer to as either *D*. cf. *micropogoni* or the new species for the NHML voucher, and these are treated below in the remarks relating to the respective descriptions.

The specimens deposited by WLB represent the only available taxonomically verifiable evidence for the presence of *D*. *leiostomi* in the Gulf of Mexico to date. A recent survey of symbionts from the bay scallop, *Argopecten irradians* (Lamarck) from the Gulf of Mexico in Florida reported metacercariae identified as *D*. *leiostomi* (Scro et al., 2023); however, these authors relied on the BLAST tool (Altschul et al., 1990) to align their generated 18S rRNA from a metacercaria with publicly available sequences and identified the material as *D*. *leiostomi* based on their sequence being closest to AY222137. Their sequence (OM680929) differs from AY222137 by 10 base substitutions over 1,830 aligned nucleotides, indicating they are probably not conspecific. Regardless, AY222137 was previously misidentified as *D*. *leiostomi*, and we herein identify that material as *D*. cf. *micropogoni* (explained in the remarks for that species below). Based on their sequence and histological photomicrograph of a metacercaria (Scro et al., 2023, Fig. 8F), which indicates presence of two testes, we agree that the metacercaria belongs in *Diplomonorchis*. However, there are five sympatric *Diplomonorchis* spp. present in their study site (*D*. *floridensis*, *D*. *leiostomi*, *D*. cf. *micropogoni*, *D*. *sphaerovarium* and the present *Diplomonorchis* **n. sp.** described below, see Nahhas & Powell, 1965; Overstreet, 1969). Four of these species lack available 18S rRNA data, so it is currently not possible to identify OM680929 to the level of species.

***Diplomonorchis*** **cf.** ***micropogoni*** **Nahhas & Cable, 1964**

**Fig. 2:**
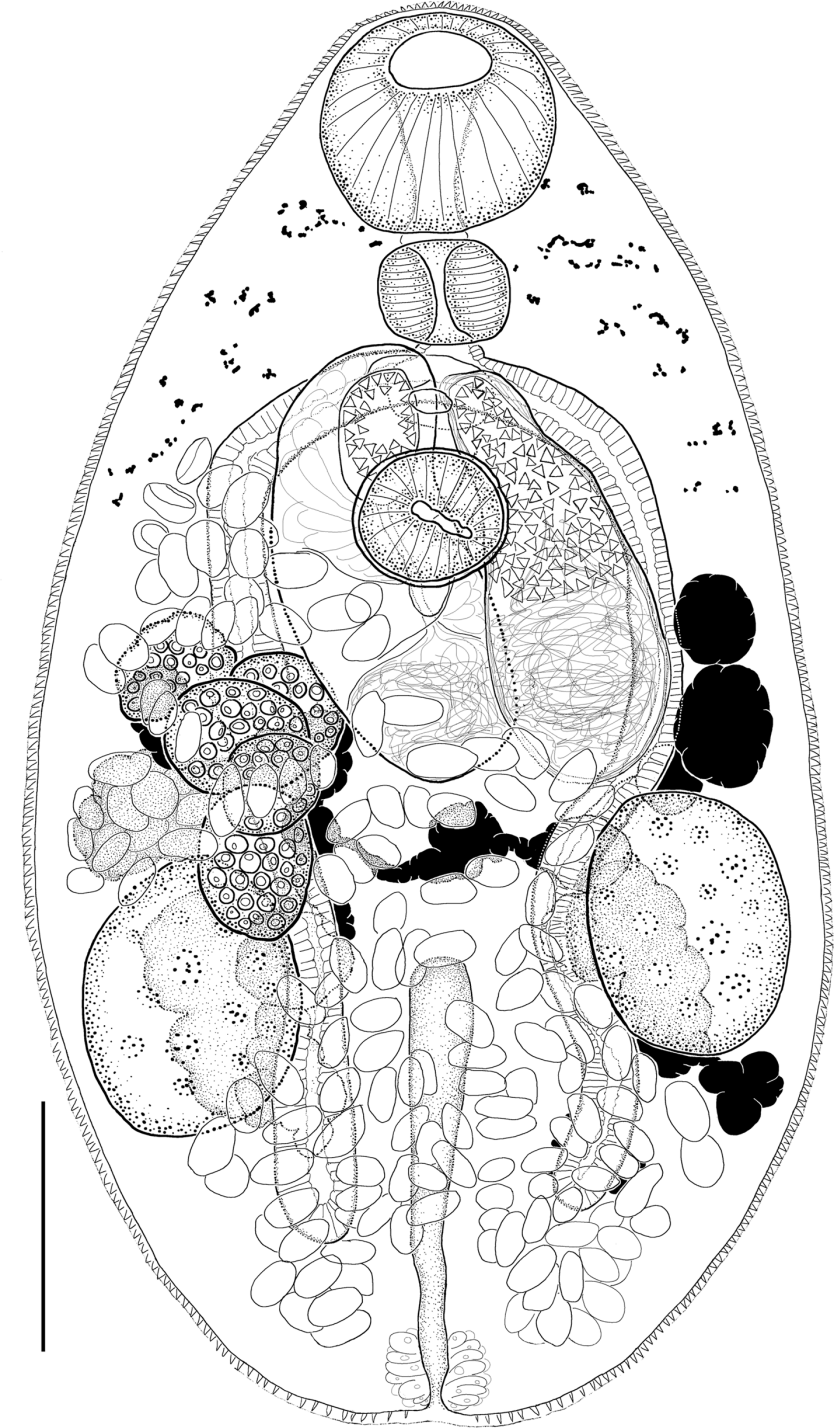
*Diplomonorchis* cf. *micropogoni*. Ventral view of adult collected from *Micropogonias undulatus* (Linnaeus) in Mississippi Sound, Gulf of Mexico. Scale bar: 100 µm.

Synonyms: *Diplomonorchis leiostomi* of Sogandares-Bernal & Hutton (1959, in part), Nahhas & Powell (1965), Overstreet (1969), Olson et al. (2003, in part), Panyi et al. (2020).

(Figs. 2, 4C; Table 2).

*Type-host: Micropogonias furnieri* (Desmarest), whitemouth croaker.

*Other host: Archosargus rhomboidalis* (Linnaeus), Western Atlantic seabream.

*Type-locality:* Jamaica.

*Other hosts and localities confirmed by museum vouchers: Archosargus rhomboidalis* from Biscayne Bay, Florida, USA (HWML 863, Overstreet, 1969, in part); *Bairdiella chrysoura* (Lacepède), silver perch, from Galveston Bay, Texas, USA (HWML 38300); *Lagodon rhomboides* (Linnaeus), pinfish, from Tampa Bay, Florida (HWML 22286, Sogandares-Bernal & Hutton, 1959, in part), North Florida Gulf Coast (USNM 1355872, Nahhas & Powell, 1965); *Leiostomus xanthurus* Lacepède, spot croaker, from coastal Mississippi, USA (NHML 2003.2.11.2b, Olson et al., 2003); *Micropogonias undulatus* (Linnaeus), Atlantic croaker, from coastal Texas, USA (USNM 1380451), coastal Mississippi, USA (USNM 1398097, 1398098); *Sciaenops ocellatus* (Linnaeus, 1766), red drum, from coastal Texas, USA (USNM 1380447).

*Host and locality of new specimens studied and vouchers deposited: Lagodon rhomboides*, from coastal Florida, USA (2 vouchers, USNM 1739552-3, 2 vouchers NHML 2024.10.4.18, 19); *Leiostomus xanthurus*, from coastal Mississippi and Alabama, USA (3 vouchers, USNM 17395554-6, 3 vouchers, NHML 2024.10.4.16, 17, 20); *Micropogonias undulatus*, from coastal Mississippi and Alabama, USA (6 vouchers, USNM 1739557-62, 4 vouchers, NHML 2024.10.4.12-15); *Stellifer lanceolatus* (Holbrook), Atlantic stardrum, from coastal Georgia, USA (1 voucher, USNM 17395563; 1 voucher NHML 2024.10.4.9).

*Site of infection:* Intestine and pyloric caeca.

*New Sequence data deposited in GenBank:* ITS1 rRNA, two isolates (PQ349805, PQ349806) from *M*. *undulatus*; ITS2 rRNA, two isolates (PQ349808, PQ349812), from *M*. *undulatus*; Partial 28S rRNA, one isolate (PQ349803) from *L*. *xanthurus*, one isolate (PQ349801) from *M*. *undulatus*.

### Supplemental data

(Based on 24 ventral mounts from *M*. *undulatus*, four ventral mounts from *L*. *xanthurus*, eight ventral and one dorsal mount from *L*. *rhomboides*). Body ovoid. Tegument spined. Mouth subterminal. Oral sucker subspherical. Ventral sucker subspherical, smaller than oral sucker, in anterior body 1/3. Prepharynx absent or very short. Pharynx subspherical. Oesophagus absent or very short. Intestine bifurcating in forebody at level closer to ventral sucker than oral sucker. Caeca, blind, extending posteriad to near mid-posttesticular zone (n=16), or to past mid-testicular zone (n=17). Forebody containing diffuse remnants of eyespots.

Testes two, ovoid, longitudinally elongate, symmetrical or slightly oblique, extracaecal, spanning middle and posterior 1/3 of hindbody. Cirrus sac claviform (120–187 µm long by 53–89 µm wide), bending, overlapping ventral sucker dorsally, extending posteriorly into ovarian zone, containing proximal ovoid internal seminal vesicle (38–82 µm long by 32–69 µm wide), narrow straight prostate duct surrounded by prostate cells, and distal cirrus. Cirrus approximately 1/3 length of cirrus sac or shorter, heavily spined; spines triangular (4–7 µm long per side).

Ovary 78–179 µm long by 62–172 µm wide, 4 or 5-lobed, dextral in anterior 1/2 of hindbody, overlapping right testis. Mehlis’ gland, Laurer’s canal, and seminal receptacle not observed. Uterus coiling throughout most of hindbody, with proximal portion descending from ovary in dextral-submedian hindbody, with coils filling intertesticular and posttesticular hindbody, ascending with coils entering slightly into ventral forebody on both sides of ventral sucker. Metraterm connecting at anterior 1/4 of terminal organ. Terminal organ (90–192 µm long by 37–93 µm wide) bipartite, nearly as large as cirrus sac; proximal portion sac-like, unspined, thin walled; distal portion flask-shaped, thick-walled, lined internally with triangular spines (6–8 µm long per side). Genital atrium median, thick-walled, lacking spines. Genital pore median in ventral forebody, at level of intestinal bifurcation (8–45 µm anteriad from ventral sucker). Vitellarium comprising 2 groups of irregular follicles. Follicles largely dorso-median to testes but extending anteriorly almost to ventral sucker and well into posttesticular zone, almost to posterior extremity.

Excretory vesicle I-shaped, extending anteriorly in dorsal hindbody to near mid-testicular level. Pore terminal.

### Remarks

Nahhas & Cable (1964) described *D*. *micropogoni* from Jamaica in the Caribbean Sea. No type-host was designated for *D*. *micropogoni* but it was originally described based on specimens collected from the whitemouth croaker, *Micropogonias furnieri* (Desmarest), and the Western Atlantic seabream, *Archosargus rhomboidalis* (Linnaeus). The holotype for *D*. *micropogoni* (USNM 1356062) is from *M*. *furnieri*, but to our knowledge no other museum voucher identified as *D*. *micropogoni* has been deposited in a lending museum. We did not examine the holotype for this work, but we examined museum vouchers identified as *D*. *leiostomi* (NHML 2003.2.11.2b; HWML 863, 22286, 38300; USNM 1380447, 1380451, 1355872) and *Diplomonorchis* sp. (USNM 1398097, 1398098) and determined that these all conformed to the original description of *D*. *micropogoni*. GenBank sequences AY222252 and AY222137 were sourced from specimens supported by the NHML vouchers as discussed above, enabling us to confidently identify all the material as conspecific. We identified the museum material in question, as well as our own new material we collected from *L*. *rhomboides*, *L*. *xanthurus*, and *M*. *undulatus*, from the Gulf of Mexico and *S*. *lanceolatus* from the Atlantic Ocean as *D*. cf. *micropogoni*. Although the specimens we examined were all indistinguishable from the original description by Nahhas & Cable (1964), we used the confer abbreviation in the name because *M*. *furnieri* was not among the hosts we collected. However, two museum vouchers from South Florida (HWML 863) were from *A*. *rhomboidalis*. Nevertheless, we suggest that our identification should be validated by sourcing nucleotide data from new specimens from *M*. *furnieri* from closer to the type locality in the Caribbean Sea.

*Diplomonorchis micropogoni* has unremarkable morphological features that make it difficult to differentiate from many of its congeners. There were only five accepted congeners when *D*. *micropogoni* was described, and Nahhas & Cable (1964) relied on three characters to distinguish it from all of them: (1) the caecal terminus being near the posterior margin of the testes; (2) the anterior extent of the uterus; and (3) egg size. Overstreet (1969) collected specimens belonging to *Diplomonorchis* from *A*. *rhomboidalis* (HWML 863), *L*. *rhomboides*, and *O*. *chrysopterus* (all from Biscayne Bay, South Florida). At that time there were eight accepted congeners (*D*. *bivitellosus*, *D*. *floridensis*, *D*. *hopkinsi*, *D*. *leiostomi*, *D*. *magnacetabulum*, *D*. *micropogoni*, *D*. *myrophitis*, and *D*. *sphaerovarium*), and the three characters used by Nahhas & Cable (1964) were no longer sufficient for delineating *D*. *micropogoni*. Overstreet (1969) used five other characters and their states when assessing the identity of his specimens: (1) relative position of the testes and vitellaria relative to mid-body; (2) whether the uterus encroaches into the forebody on one or both sides of the body; (3) whether the caeca extend into the post-testicular space or not; (4) whether spines are rarely present or always absent from the proximal portion of the terminal organ; and (5) shape and configuration of the seminal vesicle. He concluded that it was impossible to distinguish between *D*. *micropogoni* and *D*. *leiostomi* and made *D*. *micropogoni* a junior subjective synonym of *D*. *leiostomi*. He identified all his material as *D*. *leiostomi*. This action was met with universal acceptance that has endured until now.

Guided by our molecular results we revisited the task of attempting to distinguish between *D*. *micropogini* and *D*. *leiostomi* using morphological characters. We first applied comparison of the five characters used by Overstreet (1969) with our specimens of *Diplomonorchis* collected from Alabama, Mississippi, Florida and North Carolina, and museum vouchers from Texas, Mississippi, and Florida (including HWML 863). We also recovered a third slide specimen from Overstreet’s Biscayne Bay collection that lacked host identification and was morphologically identical with vouchers HWML 863. Like Overstreet (1969) we were unable to confidently differentiate *D*. *micropogoni* from *D*. *leiostomi* using these characters. Specifically, we observed that the level of the testes in the hindbody is typically in the middle third rather than the posterior third of the hindbody in both *D*. *micropogoni* and *D*. *leiostomi* but one or both testes are sometimes slightly further posterior in either species. Post-testicular space as a percentage of body length is similar for both species (19–30% in *D*. *micropogoni* verses typically 20–27% in *D*. *leiostomi*), and therefore level of the testes is ambiguous for the two species (Table 2). Similarly, the vitellaria are distributed in the gonadal region in both species and may extend beyond the gonads, either anteriorly or posteriorly, rendering this useless for distinguishing the species. While we agree with Overstreet (1969) in that there is often variation in the anterior extent of the uterus in populations of some *Diplomonorchis* spp., we believe that the variation is developmental in some species and that both* D*. *micropogoni* and *D*. *leiostomi* (as well as some other congeners - but not all) have the potential to develop uterine loops that encroach on the pharynx on either or both sides. Whether the caeca terminate near the posterior testicular margin or in the post-testicular space is often not observable in specimens due to obstruction by eggs; however, the caeca terminated in the post-testicular space in nearly all our specimens. Post-caecal space is generally longer in *D*. *micropogoni* (8–31% of body length) compared with 5–14% of body length in *D*. *leiostomi*, and with overlap notwithstanding, the caeca terminate nearer to the mid-posttesticular space than to the posterior body end in *D*. *micropogoni* and nearer to the posterior body end than the mid-posttesticular space in *D*. *leiostomi* (Table 2). The terminal organ is bipartite with a proximal spineless chamber and a distal spined portion in both* D*. *micropogoni* and *D*. *leiostomi*. The scenario alluded to by Overstreet (1969) in which perhaps a few spines are present in the proximal chamber in a species having a bipartite terminal organ is unsubstantiated and not useful for distinguishing between* D*. *micropogoni* and most congeners. Minor differences in seminal vesicle shape (e.g. size, volume) could be conditional on development or recent mating patterns and should not be relied on as a specific trait; however, we acknowledge that seminal vesicle configuration (i.e. bipartite, unipartite, coiled, sac-like) is conserved in a species. Both* D*. *micropogoni* and *D*. *leiostomi* have a near-spherical to ovoid seminal vesicle and variation between these shapes should be attributed to sperm volume and not be used to distinguish between the two species. Among these five characters only the difference at the caecal terminus was a useful character to distinguish *D*. *micropogoni* from *D*. *leiostomi*, but since that feature is often blocked from view by eggs, we refrain from relying on it as a strong feature here.

With the ability to distinguish *D*. *micropogoni* from *D*. *leiostomi* using morphology still eluding us, and since there are now 11 other accepted congeners (Table 1), we expanded our analysis to use nine collective characters and their states to attempt to distinguish *D*. *micropogoni* from its congeners: (1) the body is ovoid like in most congeners, rather than elongate (*D*. *bivitellosus*, *D*. *cumingiae*, *D*. *floridensis* and *D*. *sphaerovarium*); (2) diffuse eyespot remnants are visible in the body like in most congeners, rather than absent (*D*. *catarinensis* and *D*. *hopkinsi*), (eyespots are not reported in *D*. *alexanderi*, *D. kureh* and *D. magnacetabulum*); (3) the oral sucker is larger than the ventral sucker, rather than smaller (*D*. *magnacetabulum*), or about equal (*D*. *bivitellosus*); (4) the caeca generally terminate near the middle of the post-testicular zone (as in *D*. *caterinensis*, *D*. *cumingiae*, *D*. *hopkinsi* and *D*. *myrophitis*), rather than at the anterior margin of the testes (*D*. *alexanderi* and *D*. *kureh*), or closer to the posterior body end than the testes (*D*. *bivitellosus*, *D*. *floridensis*, *D*. *leiostomi*, *D*. *magnacetabulum* and *D*. *sphaerovarium*) (caecal terminus is indeterminate in *D*. *caballeroi*); (5) the testes are elongate, ovoid and distributed laterally and extra-caecal in the middle third of the hindbody (as in *D*. *bivitellosus*, *D*. *caballeroi*, *D*. *catarinensis*, *D*. *cumingiae*, *D*. *floridensis* and *D*. *hopkinsi*), rather than ovoid and overlapping the caeca and extending into inter-caecal space (e.g. *D*. *magnacetabulum*, *D*. *myrophitis* and *D*. *sphaerovarium*), or nearly spherical and distributed laterally and extracaecal in the middle third of the hindbody (e.g. *D*. *leiostomi*), or nearly spherical and distributed in the posterior third of hindbody (e.g. *D*. *alexanderi* and *D*. *kureh*); (6) the ovary is lobed like in most congeners rather than entire in outline (e.g. *D*. *magnacetabulum* and *D*. *sphaerovarium*); (7) the uterus circulates throughout most of the body including well into the forebody on either side of the ventral sucker and well into the post-testicular space, often obscuring the caecal terminus (like in *D*. *catarinenesis*, *D*. *hopkinsi* and *D*. *leiostomi*), rather than being mostly limited to the pretesticular space (e.g. *D*. *alexanderi*, *D*. *caballeroi* and *D*. *kureh*), or being confined to the hindbody (e.g. *D*. *bivitellosus*, *D*. *cumingiae*, *D*. *floridensis*, *D*. *magnacetabulum*, *D*. *myrophytis* and *D*. *sphaerovarium*); (8) the terminal organ is bipartite like in most congeners, rather than unipartite (e.g. *D*. *bivitellosus*, *D*. *catarinensis* and *D*. *cumingiae*); and (9) the eggs are operculated, oval, and range in size from 20–30 µm long by 11–19 µm wide (Nahhas & Cable, 1964; Table 2). Egg size of *D*. *micropogoni* overlaps with 9 of the congeners, but 3 congeners (*D*. *catarinensis*, *D*. *cumingiae*, and *D*. *hopkinsi*) have substantially smaller eggs (≤ 20 µm long by ≤ 11 µm wide) (Martin, 1940; Nahhas & Cable, 1964; Amato, 1982).

Two of the nine characters were useful for differentiating *D*. *micropogoni* from *D*. *leiostomi*: the caecal terminus that was insufficient alone, plus testes size and shape. *Diplomonorchis micropogoni* has larger, elongate ovoid testes measuring 54–166 µm long by 60–80 µm wide (Nahhas & Cable, 1964), 74–148 µm long by 49–103 µm wide (our collections from Gulf of Mexico), and 87–173 µm long by 53–97 µm wide (in 39 museum vouchers misidentified as *D*. *leiostomi* from the Gulf of Mexico). In contrast, *D*. *leiostomi* has smaller, nearly spherical testes measuring 34–72 µm long by 25–68 µm wide) (Table 2). This represents a clear metric for differentiating between *D*. *micropogoni* and *D*. *leiostomi*, and we consequently were able to identify our collected specimens and previously misidentified and unidentified museum vouchers as *D*. *micropogoni* primarily based on their testes size and shape. Hopkins (1941) noted that *D*. *leiostomi* had “more rounded” testes than *D*. *bivitellosus*, but testes size and shape has not been subsequently employed to distinguish among congeners of *Diplomonorchis*. In addition to testes size, we recognize that when the caecal terminus is observable in *D*. *micropogoni*, it occurs more frequently near the middle of the post-testicular zone. However, this overlaps with the range of caecal termination in *D*. *leiostomi* making this a poor character for delineating the two species.

***Diplomonorchis fallax*** **n. sp.**

**Fig. 3:**
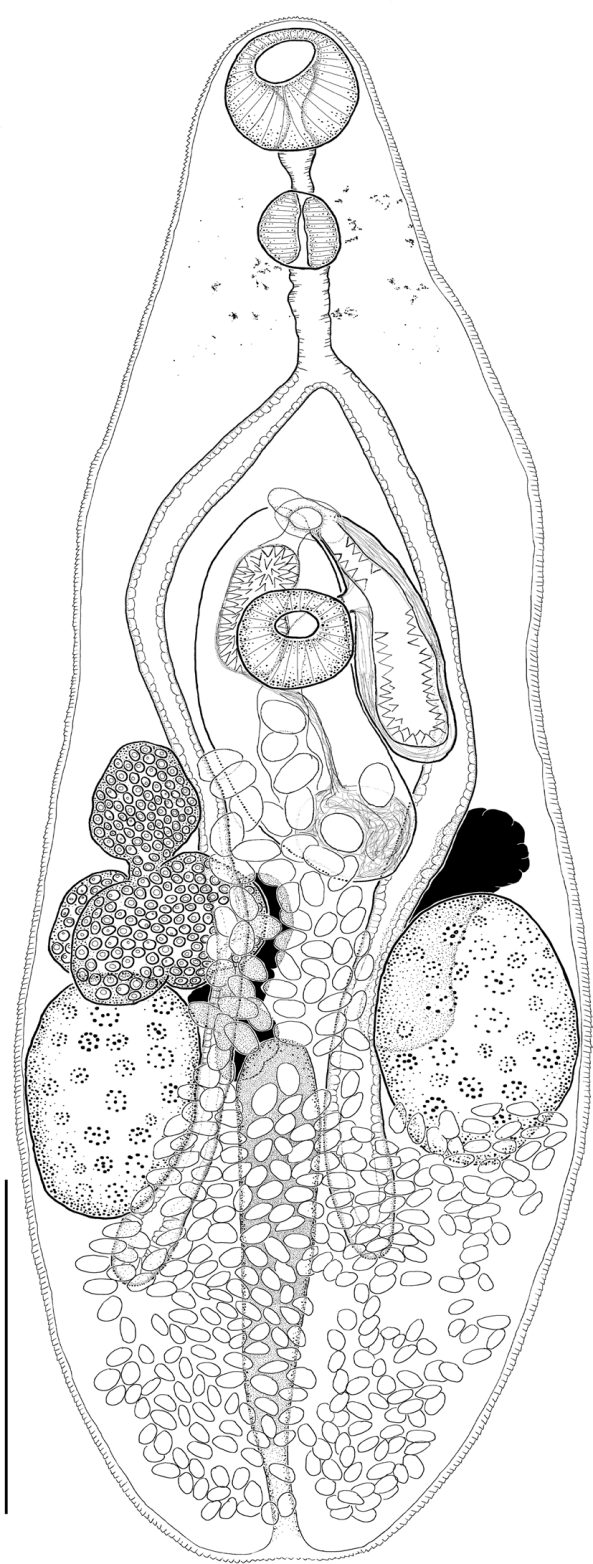
*Diplomonorchis fallax* **n. sp.** Ventral view of holotype collected from *Leiostomus xanthurus* Lacepède in Biloxi Bay, Mississippi, USA. Scale bar: 150 µm.

**Fig. 4:**
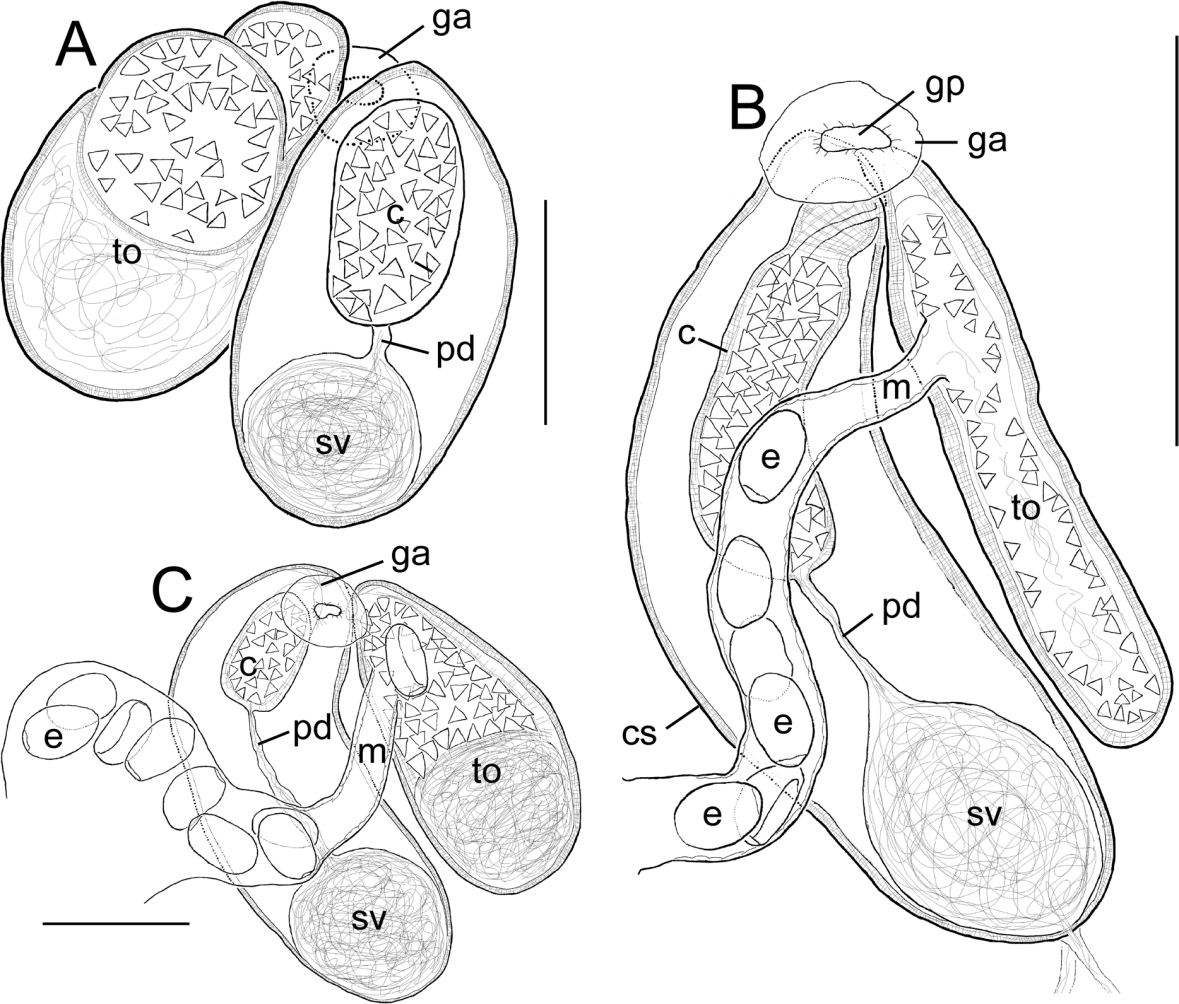
Male and female terminal genitalia from *Diplomonorchis* spp. A, Dorsal view* D*. *leiostomi*; B, Ventral view *D*. *fallax* **n. sp.**; C, Ventral view *D*. cf. *micropogoni*. Abbreviations: c, cirrus; cs, cirrus sac; e, egg; ga, genital atrium; gp, genital pore; m, metraterm; pd, prostatic duct; sv, seminal vesicle; to, terminal organ. Scale bars: A, 40 µm; B 75 µm; C, 50 µm.

Synonyms: *Diplomonorchis leiostomi* of Olson et al. (2003, in part).

(Figs. 3, 4B; Table 2).

*Type-host: Leiostomus xanthurus* Lacepède, spot croaker.

*Type-locality:* Biloxi Ship Channel, Mississippi, USA (30° 23ʹ 19.74″ N, 88° 49ʹ 33.22″ W)

*Other locality for paratypes:* Mobile Bay, Alabama, USA (30° 15ʹ 27.69″ N, 87° 53ʹ 3.27″ W).

*Other hosts and localities from museum specimens and collections: Micropogonias undulatus* (Linnaeus), Atlantic croaker (specimens collected by Robin M. Overstreet in 1970), and *L*. *xanthurus* (NHML 2003.2.11.2a), Mississippi Sound, Mississippi USA.

*Site of infection:* Intestine.

*Type-material deposited:* Holotype (USNM 1739564), 10 paratypes (USNM 1739565-74, 8 paratypes NHML 2024.10.4.1-8).

*Sequences deposited in GenBank:* ITS1 rRNA, 1 isolate (PQ349807); ITS2 rRNA, 2 isolates (PQ349813, PQ349809); partial 28S rRNA, 2 isolates (PQ349802, PQ349384).

*Etymology:* The species name *fallax* is a masculine, feminine or neuter nominative singular Latin adjective. The name means deceptive and is applied because some parasitologists have collected this species from the Gulf of Mexico and identified it as *D*. *leiostomi*. The gender of the genus name is controversial. The plant named *Orchis* Tournefort in Linnaeus, has long been treated as feminine by botanists despite *orchis* also being the Latinized Greek term for testis. Zoologists have traditionally treated genus names with the suffix *orchis* as masculine, though the plant was named first. The name *fallax* circumvents this grammatical problem regardless of how the gender for *orchis* is interpreted in zoology.

### Description

(Based on 30 ventral wholemounts, one dorsal wholemount, and one lateral wholemount, all from *L*. *xanthurus*). Body elongate-pyriform, dorsoventrally flattened, tapering toward anterior end and rounded at posterior end with a slight notch at the terminal end. Tegument spined. Mouth subterminal. Oral sucker subspherical. Ventral sucker subspherical, much smaller than oral sucker, in anterior body half. Prepharynx present, at least half length of and often longer than pharynx. Pharynx subspherical to barrel-shaped. Oesophagus almost always longer than pharynx. Intestine bifurcating midway between suckers. Caeca extending slightly into post-testicular zone, blind. Forebody containing diffuse remnants of eyespots.

Testes 2, elongate-ovoid, symmetrical or slightly oblique, extracaecal in middle 1/3 of hindbody. Cirrus sac claviform (119–220 µm long by 51–83 µm wide), bending, overlapping ventral sucker dorsally, extending posteriorly into ovarian zone, containing proximal ovoid internal seminal vesicle (41–85 µm long by 36–63 µm wide), narrow straight prostate duct surrounded by dense prostate cells, and distal cirrus. Cirrus occupying anterior 1/3 or more of cirrus sac, about twice as wide when everted than when inverted, heavily spined; spines triangular.

Ovary 83–222 µm long by 50–195 µm wide, 3 or 4-lobed, dextral in anterior 1/2 of hindbody; posterior margin adpressed with anterior margin of right testis. Mehlis’ gland, Laurer’s canal, and seminal receptacle not observed. Uterus confined to intercaecal and posttesticular space in hindbody; proximal portion descending from ovary in dextral-submedian hindbody, coiling between testes and expanding posteriad and filling posttesticular space, then ascending in sinistral-submedian or median hindbody toward metraterm in ventral forebody. Metraterm connecting to anterior 1/3 to 1/4 of terminal organ. Terminal organ muscular (92–147 µm long by 30–63 µm wide), unipartite, elongate-pyriform, with internal surface entirely spinose; proximal end wide and rounded, tapering toward narrow distal end. Genital atrium median, thick-walled, lacking spines. Genital pore median in ventral forebody, halfway between intestinal bifurcation and ventral sucker (8–44 µm anteriad from ventral sucker). Vitellarium comprising 2 groups of irregular follicles. Follicles largely dorso-median to testes but extending anteriorly into ovarian zone and slightly into posttesticular zone. Unmeasured proximal eggs larger than measured eggs from distal portion of uterus.

Excretory vesicle I-shaped, extending anteriorly in dorsal hindbody to posterior margin of ovary. Pore terminal.

### Remarks

*Diplomonorchis fallax* **n. sp.** becomes the fourteenth accepted species in *Diplomonorchis* and tenth known from the western Atlantic Ocean. The terminal organ is unipartite in *D*. *fallax*, with the lumen of the chamber lined by triangular-shaped spines (Fig. 4B). Only three congeners have a similarly configured unipartite terminal organ: *D*. *bivitellosus*, *D*. *cumingiae*, and *D*. *catarinensis* (Manter, 1940; Martin 1940; Amato, 1982). The remaining ten congeners have a bipartite terminal organ with the proximal chamber spineless and the distal chamber being lined internally with spines like in *D*. *leiostomi* and *D*. *micropogoni* (Figs. 4A, C).

*Diplomonorchis fallax* differs from *D*. *bivitellosus* by having a smaller oral sucker (46–59 µm long by 47–60 µm wide compared with 102 µm in diameter), smaller ventral sucker (31–45 µm long by 34–44 µm wide compared with 95 µm in diameter), having oral sucker width to ventral sucker width ratio of 1:0.6–0.8 rather than approximately 1:1, having long prepharynx (14–52 µm long) compared with none, having long oesophagus (27–73 µm long) compared with none, and by having a cirrus sac longer rather than equal in length to the terminal organ (Manter, 1940). *Diplomonorchis bivitellosus* was originally described infecting the halfspotted tonguefish, *Symphurus atramentatus* Jordan & Bollman from the Galápagos Islands in the Pacific Ocean (Manter, 1940). Interestingly, Pearse (1949) reported *D*. *bivitellosus* infecting the blackcheek tonguefish, *Symphurus plagiusa* (Linnaeus) from the Atlantic Ocean off Beaufort, North Carolina, USA and included a superficial illustration of a specimen depicting a worm with a ventral sucker much smaller than the oral sucker. Nahhas & Powell (1965) considered the material studied by Pearse (1949) to be identical with that named *Distomum* sp. infecting *S*. *plagiusa* at Beaufort, North Carolina, USA studied by Linton (1905). We agree with Nahhas & Powell (1965) in that the specimens from *S*. *plagiusa* studied by Linton (1905) and Pearse (1949) are probably conspecific, but we suggest they may represent *D*. *floridensis*. *Diplomonorchis floridensis*, which was described from *S*. *plagiusa* from the Gulf of Mexico in Florida, USA, has a bipartite terminal organ and the oral sucker is slightly larger than the ventral sucker rather than equal as in *D*. *bivitellosus*, but the 2 species don’t differ significantly otherwise. Both Beaufort studies lack sufficient detail for classifying the condition of the terminal organ as unipartite or bipartite, but in any case, *D*. *fallax* differs from the nebulous Beaufort material by having smaller suckers: oral sucker 46–59 µm long by 47–60 µm wide compared with 90–160 in diameter; ventral sucker 31–45 µm long by 34–44 µm wide compared with 90–150 in diameter; and anterior extent of vitelline follicles limited to ovarian region rather than extending into the level of the ventral sucker (Linton, 1905; Pearse, 1949).

*Diplomonorchis fallax* differs from *D*. *cumingiae* by having a larger body (body 526–879 µm long by 193–305 µm wide compared with 255–318 µm long by 100–164 µm wide), relatively shorter forebody (34% of body length compared with 38%), longer prepharynx (14–52 µm long compared with 3–4 µm), larger pharynx (28–39 µm long by 28–36 µm wide compared with 19 µm long by 22 µm wide), and a longer oesophagus (27–73 µm long compared with ~5 µm) (Martin, 1940). *Diplomonorchis cumingiae* was originally described based on larval material from a tellinoid clam (*Cumingia sinuosa* A. Adams) at Woods Hole, Massachusetts, USA (Martin, 1938). In a subsequent life cycle investigation Martin (1940) determined that unidentified eels and flounders served as the natural definitive hosts for the species in the vicinity of Woods Hole. Adult specimens of *D*. *cumingiae* have not been reported on since and the identity of the definitive hosts remains ambiguous.

*Diplomonorchis fallax* resembles *D*. *catarinensis* more closely than all other congeners. Nevertheless, *D*. *fallax* differs from *D*. *catarinensis* by being slightly larger in body size (526–879 µm long by 193–305 µm wide compared with 408–571 µm long by 112–204 µm wide), having a more elongate, pyriform shaped body rather than an oval shaped body, having rather than lacking eyespot pigment in the anterior body half, having a smaller oral sucker (46–59 µm long by 47–60 µm wide compared with 60–64 µm long by 60–72 µm wide), having a longer prepharynx (14–52 µm compared with 6–12 µm long), and having a smaller ventral sucker (31–45 µm long by 34–44 µm wide compared with 42–50 µm long by 44–58 µm wide) (Amato, 1982). Additionally, the uterus is confined to the hindbody rather than having lateral coils extend into the forebody, the genital pore is well-posterior from, rather than at the intestinal bifurcation (which is probably a manifestation of having a more elongate body), and the eggs are slightly larger 17–23 µm long by 11–14 µm wide compared with 18–20 µm long by 7–10 µm wide (Amato, 1982). *Diplomonorchis catarinensis* was described from *M*. *furnieri* and the Atlantic spadefish, *Chaetodipterus faber* (Broussonet) from the southern Atlantic Coast of Brazil. It has not been subsequently reported from either host to our knowledge. We have examined many specimens of *C*. *faber* for parasites in the northern Gulf of Mexico and not collected *D*. *catarinensis*, and we are not aware of any reports of monorchiids from this host from the Gulf of Mexico (Overstreet et al., 2009).

### Phylogenetic analysis

**Fig. 5:**
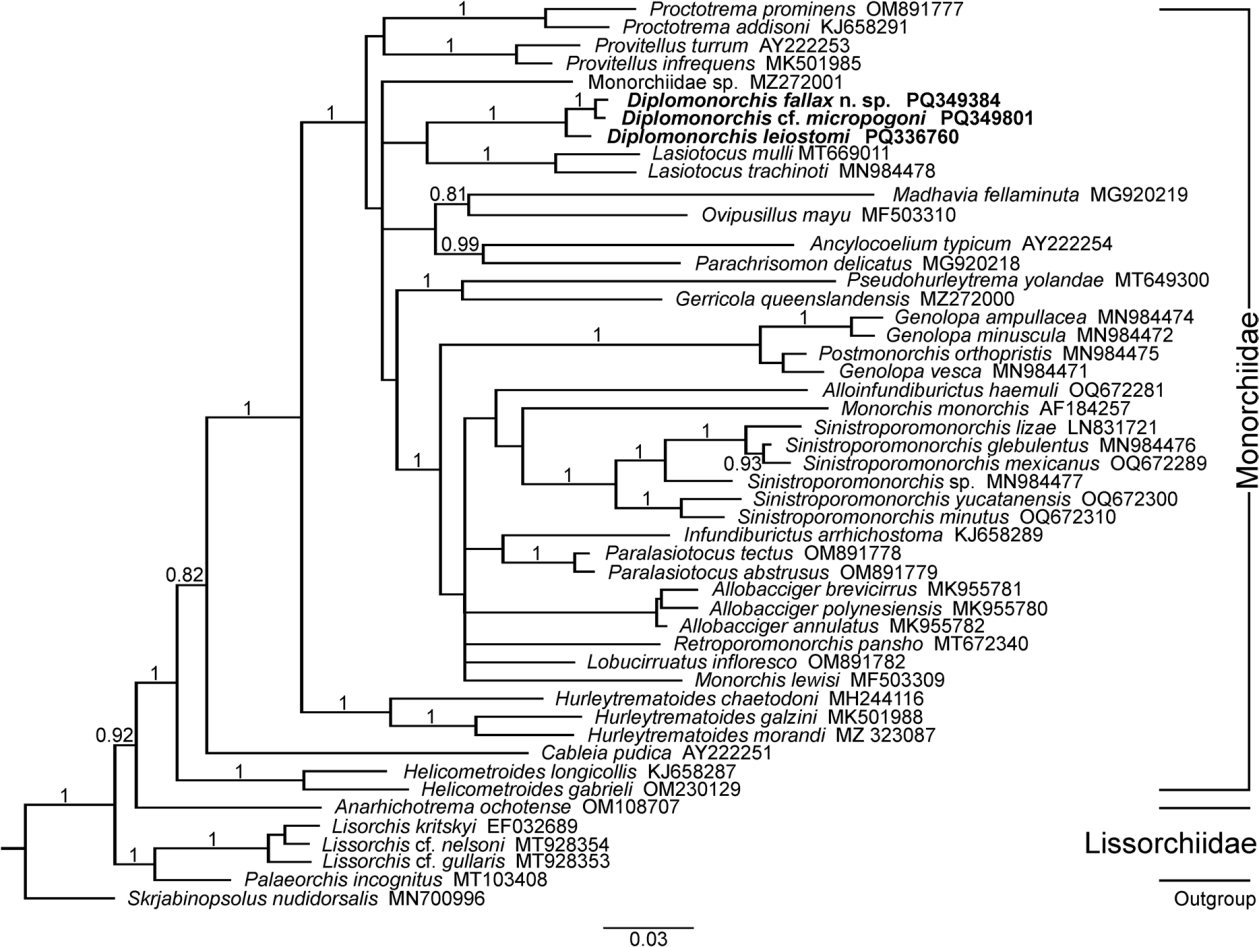
Phylogram estimating the relationships of *Diplomonorchis* spp. among some of the Monorchioidea Odhner, 1911 based on Bayesian Inference analysis of partial 28S rRNA sequences. Posterior probabilities are provided at nodes when greater than 80%. *Skrjabinopsolus nudidorsalis* Sokolov, Voropaeva, & Atopkin, 2020 (Monorchioidea: Deropristidae) serves as the outgroup. Scale bar indicates substitutions per site.

The phylogenetic tree from the analysis (Fig. 5), constructed entirely of species from the Monorchioidea, exhibits similar topology with previous phylogenetic studies centering on the Monorchiidae and related families (Wee et al. 2018; Sokolov et al., 2020; Wee et al., 2020a, 2020b, 2020c, 2021, 2022). The basal nodes of the tree are all well-supported. The Lissorchiidae are firmly basal to the Monorchiidae as demonstrated in Sokolov et al. (2020). The basal nodes within the Monorchiidae are dominated by genera classified in the Hurleytrematinae Yamaguti, 1958 with the enigmatic *Cableia* Sogandares-Bernal, 1959 nested between species in the more basal *Helicometroides* Yamaguti, 1934 and species in the more derived *Hurleytrematoides* Yamaguti, 1954 as previously demonstrated (Wee et al., 2018, 2020a, 2020b, 2020c, 2021). The basal portion of the main monorchid clade is unresolved and contains both the hurleytrematine *Provitellus* Dove & Cribb, 1998 and monorchiine *Proctotrema* Odhner, 1911. The most derived group of the Monorchiidae contains 33 taxa, which are largely unresolved but contain mostly species currently classified in the Monorchiinae Odhner, 1911. Exceptionally, the enigmatic *Parachrisomon* Madhavi, 2008 and the hurleytrematine, *Pseudohurleytrema* Yamaguti, 1954 are nested among monorchiine taxa. The 3 studied species of *Diplomonorchis* form a poorly supported relationship with 2 species of *Lasiotocus* Looss, 1907, including the type-species *Lasiotocus mulli* (Stossich, 1883) Odhner, 1911. Some previous studies examining interrelationships among the Monorchiidae that utilized *D*. cf. *micropogoni* (AY222252) demonstrated a similarly unresolved relationship for *Diplomonorchis* with an affinity for some species of *Lasiotocus* (Sokolov et al., 2020; Wee et al., 2020a, Wee et al., 2020b, Wee et al., 2021, Wee et al., 2022).

## Discussion

Taxonomists concerned with monorchiid taxonomy from the warm western Atlantic Ocean have consistently embraced a broad concept of the first described species of *Diplomonorchis*, *D*. *leiostomi*. This study provides an alternative perspective on the identification of *D*. *leiostomi*, restores *D*. *micropogoni*, and distinguishes *D*. *fallax* as a third species previously mistaken as a morphological variant confused with *D*. *leiostomi* and *D*. *micropogoni* by some workers in the northern Gulf of Mexico. Some misconceptions by previous workers may stem from an overlapping and Catholic host preference exhibited by these taxa. Based on studies by Hopkins (1941), Thoney (1991, 1993) and our observations, we confirmed that *D*. *leiostomi* infects at least *L*. *xanthurus*, *M*. *undulatus* (both Sciaenidae) and *O*. *chrysoptera* (Haemulidae). Based on the study by Nahhas & Cable (1964) and our observations of museum specimens and new collections we confirmed that *D*. *micropogoni* infects *B*. *chrysura*, *L*. *xanthurus*, *M*. *undulatus*, *M*. *furnieri*, and *S*. *lanceolatus* (all Sciaenidae), and *A*. *rhomboidalis*, and *L*. *rhomboides* (both Sparidae). We described *D*. *fallax* from *L*. *xanthurus* but we also inherited hundreds of slide mounted specimens of *D*. *fallax* collected from *M*. *undulatus* from Mississippi, USA by Robin M. Overstreet in 1970. We refrained from using these specimens in the description because they are in poor condition and lack detailed metadata. Regardless, *D*. *leiostomi* and *D*. *micropogoni* exhibit euryxenous host specificity (i.e. they infect more than one family of hosts), and *D*. *fallax* is at least mesostenoxenous (i.e. infecting at least two species of hosts from a family) (Caira et al., 2003).

The key features useful for distinguishing *D*. *leiostomi* from *D*. *micropogoni* are here established as testes size, testes shape, and to a minor degree, relative distance between the caecal terminus and the posterior body end. *Diplomonorchis leiostomi* has relatively small testes (ranging from 32–77 µm long by 25–68 µm wide) that are close to spherical in shape (ratio of length to width 1:0.7–1.2) and the caecal terminus is usually between the middle of the post-testicular space and the posterior end of the body. *Diplomonorchis micropogoni* has relatively larger testes (ranging from 54–174 µm long by 38–103 µm wide) that are longitudinally elongate (ratio of length to width 1:0.4–0.8), and the caecal terminus is usually near the middle of the post-testicular space. *Diplomonorchis fallax* is distinct from both *D*. *leiostomi* and *D*. *micropogoni* by having an elongate rather than oval body, presence of a distinct prepharynx that is usually as long as and often longer than the pharynx (rather than having the prepharynx very short or absent), presence of a long oesophagus (usually twice as long or longer than in *D*. *leiostomi* and *D*. *micropogoni*), uterus is confined to the hindbody rather than extending into the forebody, and by having smaller eggs (17–23 µm long by 11–16 µm wide) compared with eggs ranging from 20–31 µm long by 11–21 µm wide collectively in the 2 congeners. While *D*. *fallax* has large, elongate testes (ratio of length to width 1:0.4–0.8) like *D*. *micropogoni*, its suckers are relatively small like in *D*. *leiostomi*.

The present phylogenetic analysis confirms a close relationship among the three studied *Diplomonorchis* spp. but otherwise provides no new insight into interrelationships among genera than in recent phylogenetic studies focused on the Monorchiidae (Panyi et al., 2020; Sokolov et al., 2020; Wee et al., 2020a, 2020b, 2020c, Wee et al., 2021, Wee et al., 2022). Further progress at understanding generic affinities between and within the Hurleytrematinae and Monorchiinae requires the acquisition of more generic taxa that are currently not represented by 28S rRNA sequence data, as advocated by Wee et al. (2021).

The internal transcribed spacer regions have long been investigated as taxonomic markers for digeneans (Nolan & Cribb, 2005; Olson & Tkach, 2005). Nolan & Cribb (2005) summarized results from 14 early studies that investigated the entire ITS region in various digenean species; 6 of the 14 reported no evidence of intraspecific variation; 4 reported intraspecific variation of less than 1%, and the other 4 reported intraspecific variation between 1.2–3.2%. Additionally, Nolan & Cribb (2005) summarized data from 44 studies that investigated variation in the ITS2 region in digeneans and only 16 of these reported any intraspecific variation which ranged from 0.3–3.5%. The absence of, or low rates of, intraspecific variation typically exhibited in the ITS regions across most digenean taxa has led to the ITS regions being promoted as effective species-level markers, particularly the ITS2 region, which lacks variously long tandem nucleotide repeats that are often present in the ITS1 region in certain digenean families (Nolan & Cribb, 2005). The ability to rely on a species-level marker, such as the ITS2, to consistently identify larval forms by matching their DNA with DNA from adult forms is invaluable for ecological studies (Cribb et al., 1998; Galaktionov et al., 2012; Fayton et al., 2016; Gilardoni et al., 2020; Hill-Spanik et al., 2021; Curran et al., 2022). Likewise, the conserved nature of the ITS2 region, at the species level, makes it highly useful for taxonomic studies focused on distinguishing closely related digenean congeners (see Tkach et al., 2000; Parker et al., 2010; Curran et al., 2018), or identifying a species that occurs across a large geographical region (see Adlard et al., 1993; Lo et al., 2001; Huang et al., 2004). Rarely though, closely related congeners may have identical ITS regions yet still be recognized as distinct species. Nolan & Cribb (2005) highlighted three such studies: one involving two morphologically distinguishable congeneric species of *Diplostomum* von Nordmann, 1832 having identical ITS1 regions (see Niedwiadomska & Laskowski, 2002); a second in which three species of *Schistosoma* Weinland, 1858 possibly share identical ITS2 regions (see Despres et al., 1992; Nolan & Cribb, 2005); and a third in which morphologically distinct species of *Paragonimus* Braun, 1899, one from Japan and the other from China, have identical ITS2, but variation at the mitochondrial cox1 gene was used to delineate the species (Blair et al., 1997). Blair et al. (2005) expanded the earlier study and reconsidered mitochondrial cox1 gene results and preferred to synonymize the previously distinguished species as a subspecies of a *Paragonimus skrjabini* Chen, 1959 complex. Nevertheless, instances where different congeneric digenean species exhibit identical ITS1 or ITS2 regions are exceedingly rare.

Advocates of integrative taxonomic approaches have espoused the blending of morphological observations with multi-loci analyses rather than relying on either morphology alone or the ITS region alone for differentiating digenean taxa (Otranto et al., 2007; Nadler & Pérez-Ponce-de León, 2011; Blasco-Costa et al., 2016). Some of our present data (morphological features and 1.4% divergence of 28S rRNA sequences) demonstrate clear interspecific variation between *D*. cf. *micropogoni* and *D*. *fallax*. On the other hand, genetic variation in the ITS1 rDNA region (1.2% divergence between the 2 species) lies close to the interface between what can be considered intraspecific and interspecific variation in studied digeneans (Nolan & Cribb, 2005). Surprisingly, there is no variation in the ITS2 rDNA region between the species. Nevertheless, we consider these distinct species based on morphology, 28S and ITS1 rDNA, and are not cryptic species in the strict sense as they can be differentiated solely on morphological features. The absence of variation at the ITS2 region underscores the importance of analyzing at least two or more nuclear gene regions from a digenean. Particularly because reliance on the ITS2 region alone to identify larval stages in bivalves would clearly not be effective in this case. We did not employ analysis of mitochondrial genes here, which can be effective tools for investigating close relationships, because data from more *Diplomonorchis* spp., or data from a particular *Diplomonorchis* sp. collected over a large geographic area are presently unavailable and are needed to assess natural population-level mitochondrial gene variation in species of the genus.

## Data Availability

Type and voucher materials of specimens studied have been deposited in publicly accessible museums. Representatives of the generated sequences are uploaded in GenBank. Additional specimens not deposited are available by request from the authors.
